# Neutrophils Induced Licensing of Natural Killer Cells

**DOI:** 10.1155/2015/747680

**Published:** 2015-04-21

**Authors:** Keishiro Amano, Masahiro Hirayama, Eiichi Azuma, Shotaro Iwamoto, Yoshitaka Keida, Yoshihiro Komada

**Affiliations:** ^1^Department of Pediatrics, Mie University Graduate School of Medicine, 2-174 Edobashi, Tsu, Mie 514-8507, Japan; ^2^Department of Cell Transplantation, Mie University Graduate School of Medicine, 2-174 Edobashi, Tsu, Mie 514-8507, Japan

## Abstract

Natural killer (NK) cells acquire effector function through a licensing process and exert anti-leukemia/tumor effect. However, there is no means to promote a licensing effect of allogeneic NK cells other than cytomegalovirus reactivation-induced licensing in allogeneic hematopoietic stem cell transplantation in human. In mice, a licensing process is mediated by Ly49 receptors which recognize self-major histocompatibility complex class I. The distribution of four Ly49 receptors showed similar pattern in congenic mice, B10, B10.BR, and B10.D2, which have B10 background. Forty Gy-irradiated 2 × 10^6^ B10.D2 cells including splenocytes, peripheral blood mononuclear cells in untreated mice, or granulocyte colony-stimulating factor treated mice were injected intraperitoneally into B10 mice. We found that murine NK cells were effectively licensed by intraperitoneal injection of donor neutrophils with its corresponding NK receptor ligand in B10 mice as a recipient and B10.D2 as a donor. Mechanistic studies revealed that NK cells showed the upregulation of intracellular interferon-*γ* and CD107a expression as markers of NK cell activation. Moreover, enriched neutrophils enhanced licensing effect of NK cells; meanwhile, licensing effect was diminished by depletion of neutrophils. Collectively, injection of neutrophils induced NK cell licensing (activation) via NK receptor ligand interaction.

## 1. Introduction

Allogeneic hematopoietic stem cell transplantation (HSCT) is a well-established therapy for a variety of malignant disorders. Unfortunately, some patients may relapse, but they may potentially have the benefit of graft-versus-leukemia (GVL) or graft-versus-tumor (GVT) effect [1, 2]. There may be several kinds of effectors in GVL/GVT. Among them, T cell-mediated GVL/GVT effect might be potent. However, alloreactive natural killer (NK) cells display GVL/GVT, which is increasingly being recognized as an important component of the overall antileukemia/tumor effect in HSCT [2, 3]. The expansion and persistence of educated (licensed) NKG2C^+^ NK cells were found after cytomegalovirus reactivation in patients receiving allogeneic HSCT [4]. Recent murine HSCT studies suggest that maximal effect of antileukemia is dependent on whether alloreactive NK cells are licensed. Indeed, a licensing effect of NK cells is driven by the interaction of Ly49H with murine cytomegalovirus-encoded protein m157 [5]. However, cytomegalovirus infection is a potentially life-threatening complication [6, 7]. There are no reported methods for inducing a licensing effect of NK cells safely.

Neutrophils play an essential role in the body's first line of defense against bacterial and fungal infections. Jaeger et al. described that neutrophil-induced NK cell maturation may occur not only in the bone marrow where NK cells develop but also at the periphery where direct NK cells/neutrophils interaction takes place in lymph nodes and spleen [8]. The ability of NK cells to form conjugates with neutrophils revealed the strong propensity of these two cell types to interact. Thus, they suggested a new role for neutrophils as nonredundant regulatory cells ensuring the terminal maturation of NK cells. However, the precise mechanism by which neutrophils participate in NK cell maturation is still to be determined. We have pursued a mechanistic interpretation of neutrophil-induced NK cell maturation.

NK cells are thought to recognize missing self, the lack of normal expression of major histocompatibility complex (MHC) class I molecule [9]. Murine NK cells express inhibitory receptors of the Ly49 C-type lectin superfamily interacting with H-2. NK cells require engagement of an inhibitory receptor with MHC class I to attain functional competence. This process, termed licensing, allows NK cells to be activated through activation receptors to detect and kill cells lacking self-MHC class I [9]. NK cells without self-MHC-specific inhibitory receptors remain unlicensed and hence are unable to react against MHC class-I-deficient cells, thus avoiding autoreactivity. Therefore, the NK cell inhibitory receptors have a second function in licensing of NK cells in self-tolerance. In the current study, we have analyzed whether neutrophils promote a licensing effect of NK cells by its corresponding NK receptor ligand. Our results suggest that NK cell licensing by neutrophils is working in mice.

## 2. Materials and Methods

### 2.1. Mice

C57BL/10 Sn (B10, H-2^b^), B10.D2/nSn (H-2^d^), B10.BR/Sg Sn (H-2^k^), DBA/2 Cr (H-2^d^), C3H/HeJ (H-2^k^), and BALB/c Cr (H-2^d^) female mice were purchased from Japan SLC (Shizuoka, Japan). These mice, aged 8–12 weeks, were used for all experiments. The care and breeding of animals was in accordance with institutional guidelines [10]. All procedures used in this research were approved by the Ethical Committee (Permission number 24-53), Mie University Graduate School of Medicine.

### 2.2. *In Vitro* and* In Vivo* Induction of NK Cell Licensing

For* in vitro* induction of NK cell licensing, mixed lymphocyte culture was set up in 24-well plates (BD Falcon, Bedford, MA) as described previously [11]. PBMCs from B10 mice were stimulated with forty Gy-irradiated PBMCs from B10.D2 female mice. Plates were incubated at 37°C with 5% CO_2_ for 5 days before analyzing of NK cell activation markers, interferon (IFN)-*γ*, and CD107a expression.

For* in vivo* induction of NK cell licensing, forty Gy-irradiated 2 × 10^6^ splenocytes, peripheral blood mononuclear cells (PBMCs) of untreated-, or recombinant granulocyte colony-stimulating factor (G-CSF, BioLegend, San Diego, CA) treated- (subcutaneously, 250 *μ*g/mL/day, 5 days) [12] B10.D2 mice were injected intraperitoneally into B10 mice. Before and after injection of cells, PBMCs of recipient B10 mice were analyzed. In these experiments, we used splenocytes and G-CSF-treated peripheral blood. Splenocytes were isolated by Histopaque 1077 (Sigma-Aldrich, St. Louis, MO) and PBMCs were isolated by RBC Lysing Buffer (BD Biosciences, San Jose, CA). We performed experiments using enriched or depleted neutrophils for evaluation of NK licensing by neutrophils. Peripheral blood neutrophils were enriched by using the Mouse Neutrophil Enrichment Kit (Veritas Co., Ltd., Tokyo) according to the manufacturer's instruction. Peripheral blood was depleted of neutrophils by the Mouse Anti-Ly-6G Microbeads Kit (Miltenyi Biotec Inc., CA).

### 2.3. Flow Cytometry

Cells were stained with fluorescein isothiocyanate- (FITC-) conjugated monoclonal antibody (mAb) to mouse anti-Ly49A, Ly49C/I, Ly49D, Ly49G2, or Gr-1, phycoerythrin- (PE-) conjugated mAb to CD107a, IFN-*γ*, or CD11b, Allophycocyanin- (APC-) conjugated mAb to DX5 (NK cells), and Peridinin-Chlorophyll Protein Complex- (PerCP-) conjugated mAb to CD3 (BD Biosciences). For degranulation measurement, anti-CD107a mAbs were added. After an hour of incubation, monensin and brefeldin A were added. After another five hours of incubation, cells were stained with mAb. For intracellular cytokine staining, cells were fixed and permeabilized by IntraStain (Dako, Denmark), followed by antibody staining for intracellular IFN-*γ*. Fluorescence staining was analyzed with a FACSCalibur flow cytometer and the CELLQuest Software program (BD Immunocytometry Systems, San Jose, CA).

### 2.4. Statistical Analysis

Unpaired 2-tailed Student's *t*-tests were used to compare differences between groups. A *P* value less than 0.05 was considered statistically significant.

## 3. Results

**Figure 1:**
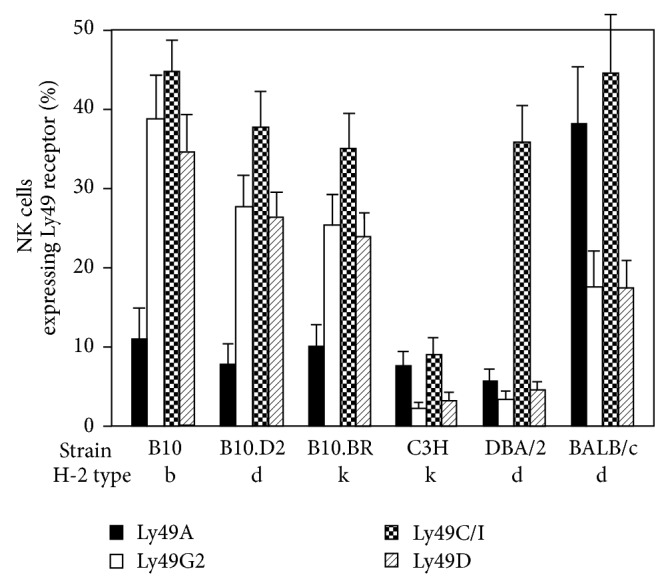
Distribution of Ly49 receptors in NK cells in different strains of mice. Freshly isolated splenocytes from B10 (*n* = 6), B10.D2 (*n* = 8), B10.BR (*n* = 7), C3H (*n* = 5), DBA/2 (*n* = 8), and BALB/c (*n* = 3) were evaluated by flow cytometer. We compared expression of Ly49 receptors on NK cells in different strains of mice. Ly49 receptors included Ly49A (its ligands are H-2^d^ and H-2^k^), Ly49G2 (H-2^d^), Ly49C/I (H-2^b^), and Ly49D (H-2^k^). Data are expressed as the means ± SD.

Ly49 receptors, binding to specific types of H-2, are primarily restricted to NK cells. To examine which combination of mice is suitable to test a NK cell licensing effect, we first analyzed the expression of Ly49 receptors, such as inhibitory Ly49A (its ligands are H-2D^d^ and D^k^), Ly49G2 (H-2D^d^), Ly49C/I (H-2K^b^), and activatory Ly49D (H-2D^d^), in various strains of mice. The distribution of four Ly49 receptors on splenic NK cells varies considerably among different strains of mice, B10, C3H, DBA/2, and BALB/c, irrespective of its corresponding ligand, H-2 (Figure 1). However, the distribution of four Ly49 receptors showed similar pattern in congenic mice, B10, B10.D2, and B10.BR, which have B10 background. Thus, these data suggest that the regulation of NK receptors resides outside MHC class I, H-2. These results were supported by the previous reports that there was no cosegregation of Ly49 family and its ligand, H-2, on different chromosomal location [13]. Based on these results, we chose B10 and B10.D2 to test NK cell licensing because these two mice with different H-2 have similar distribution of Ly49 receptors.

**Figure 2:**
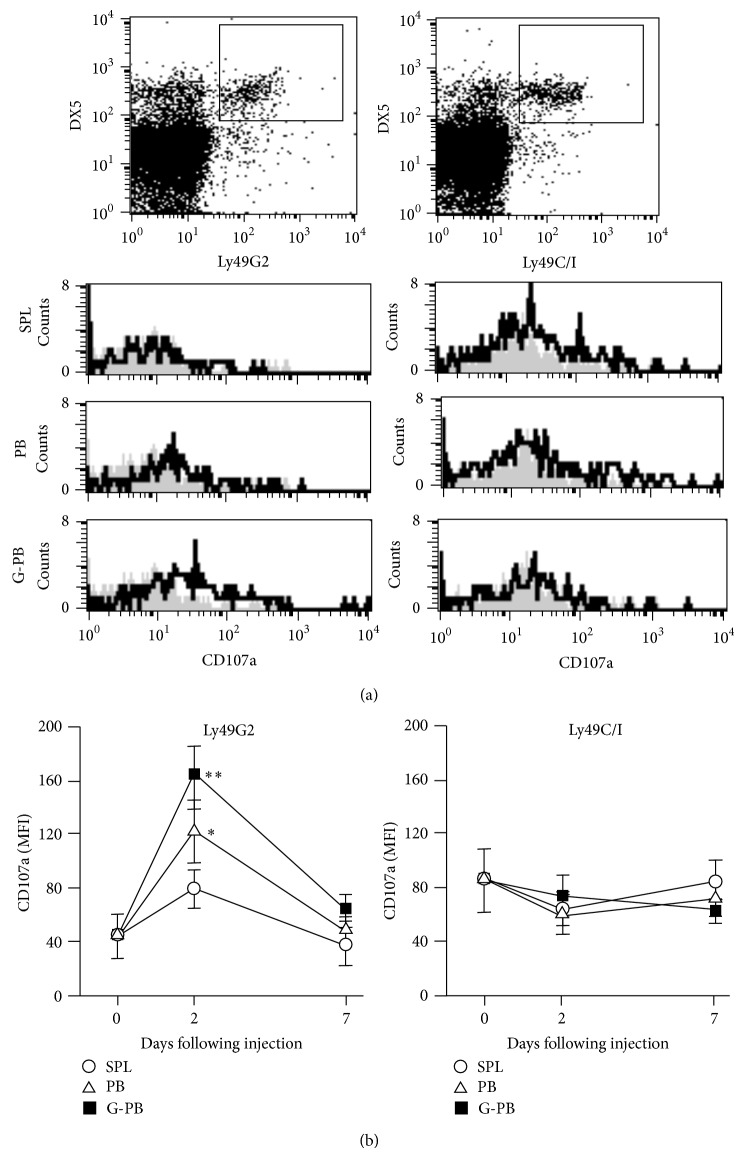
Neutrophils promote a licensing effect of NK cells: CD107a degranulation assay. Forty Gy-irradiated 2 × 10^6^ mononuclear cells (splenocyte, SPL; peripheral blood, PB; and G-CSF-treated PB, G-PB) of B10.D2 mice were injected intraperitoneally into B10 mice. Two days after injection of cells, peripheral blood mononuclear cells of recipient B10 mice were analyzed by flow cytometry. Representative histograms show CD107a expressions gated on DX5^+^CD3^−^Ly49G2^+^ cells ((a), left) and DX5^+^CD3^−^Ly49C/I^+^ cells ((a), right) in treated B10 mice (solid line) and untreated as controls (shadow area). Time course of mean fluorescence intensity (MFI) of CD107a expressions of DX5^+^CD3^−^Ly49G2^+^ cells ((b), left) and DX5^+^CD3^−^Ly49C/I^+^ cells ((b), right) was analyzed before (*n* = 11) and after injection of SPL (open circle, *n* = 18), PB (open triangle, *n* = 18), and G-PB (filled square, *n* = 8). Data are expressed as the means ± SD. ^∗^ *P* < 0.05; ^∗∗^ *P* < 0.01.

**Figure 3:**
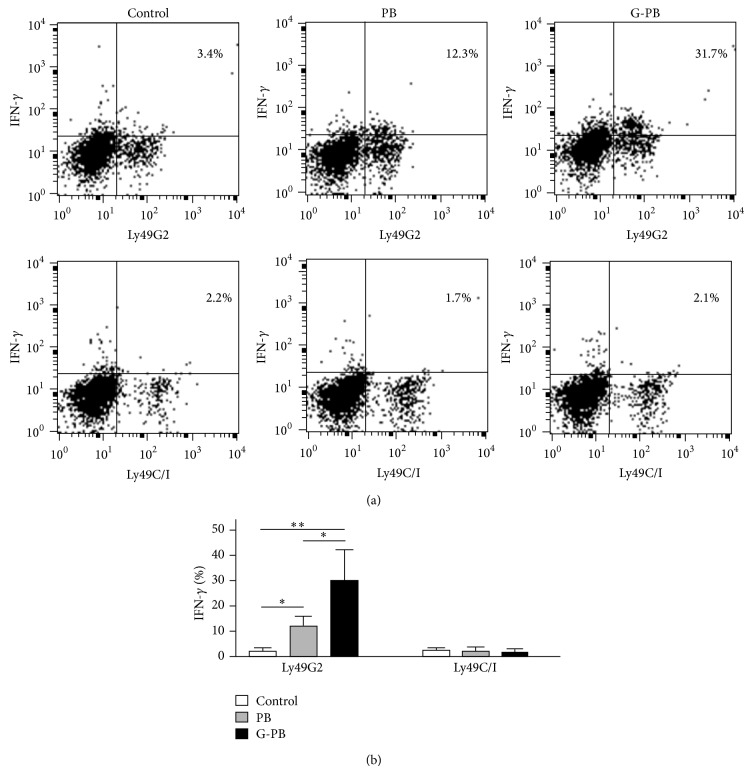
Neutrophils promote a licensing effect of NK cells: upregulation of intracellular IFN-*γ* expression. Forty Gy-irradiated 2 × 10^6^ mononuclear cells (PB and G-PB) of B10.D2 mice were injected intraperitoneally into B10 mice. Two days after injection, PBMCs of recipient B10 mice were analyzed by flow cytometry. Representative dot plots show intracellular IFN-*γ* expressions gated on DX5^+^CD3^−^Ly49G2^+^ NK cells ((a), upper panel) and DX5^+^CD3^−^Ly49C/I^+^ NK cells ((a), lower panel) in uninjected B10 PBMCs (control), B10 PBMCs injected by B10.D2 PBMCs (PB), and B10 PBMCs injected by G-CSF-treated B10.D2 PBMCs (G-PB). The comparison of IFN-*γ* expression of DX5^+^CD3^−^Ly49G2^+^ NK cells and DX5^+^CD3^−^Ly49C/I^+^ NK cells in control (*n* = 4), PB (*n* = 4), and G-PB (*n* = 4) was depicted (b). Data are expressed as the means ± SD. ^∗^ *P* < 0.05; ^∗∗^ *P* < 0.01.

Next, we have investigated whether neutrophils promote a licensing effect of NK cells by its corresponding NK receptor ligand. We used B10 mice (H-2^b^) as a recipient and B10.D2 (H-2^d^) as a donor. B10.D2 are different from B10 only in the expression of H-2 antigens. Ly49G2^+^NK cells but not Ly49C/I^+^NK cells of B10 mice were successfully licensed 2 days after intraperitoneal injection of B10.D2 cells by degranulation assay (Figure 2). The degree of surface expression of CD107a, a marker of NK cell functional activity, in Ly49G2^+^NK cells, but not in Ly49C/I^+^NK cells, was significantly proportional to a neutrophil input dose: neutrophils constituted approximately 3% of splenocytes (*n* = 18), 23% of PBMCs in untreated mice (*n* = 18), and 82% of PBMCs in G-CSF-treated mice (*n* = 8). In addition, the expression of IFN-*γ* in Ly49G2^+^NK cells but not Ly49C/I^+^NK cells of B10 mice at day 2 after intraperitoneal injection of B10.D2 PBMCs in untreated mice (*n* = 4) or G-CSF-treated mice (*n* = 4) was significantly elevated (Figure 3). The level of IFN-*γ* expression was significantly higher in the group injected with neutrophil-rich PBMCs (*P* < 0.01, Figure 3(b)). These results suggest that NK cells can be specifically licensed by presentation of its corresponding NK receptor ligand of neutrophils.

**Figure 4:**
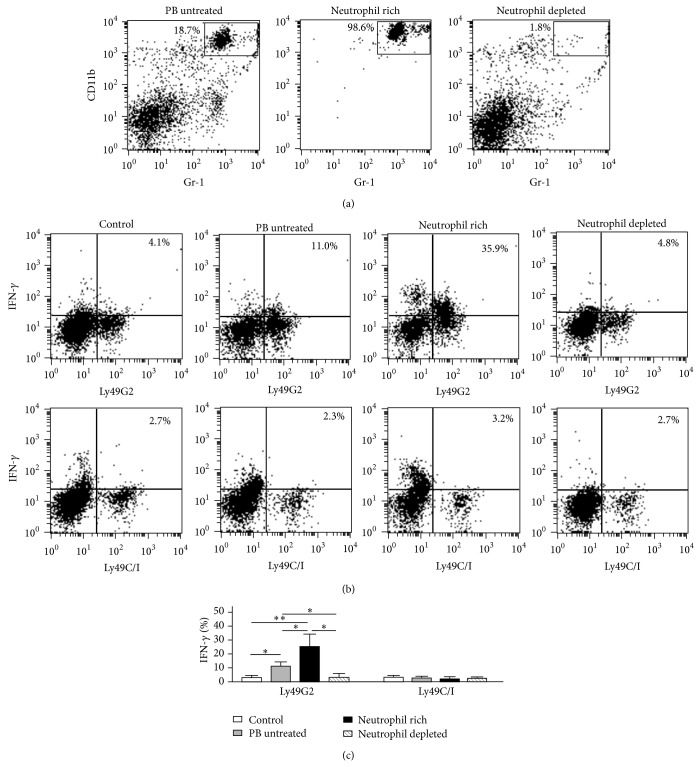
Enrichment and depletion of neutrophils affect licensing effect of NK cells. B10.D2 PB was enriched or depleted of CD11b^+^Gr-1^+^ neutrophils by magnet beads negative selection (a). Forty Gy-irradiated 2 × 10^6^ PBMCs or neutrophil-enriched/depleted cells were injected intraperitoneally into B10 mice. Two days after injection, PBMCs of recipient B10 mice were analyzed by flow cytometry. Representative dot plot analysis showed intracellular IFN-*γ* expressions gated on DX5^+^CD3^−^Ly49G2^+^ NK cells ((b), upper panel) and DX5^+^CD3^−^Ly49C/I^+^ NK cells ((b), lower panel) in uninjected B10 PBMCs (control), B10 PBMCs injected by B10.D2 PBMCs (PB untreated), B10.D2 PBMCs enriched neutrophils (neutrophil rich), and B10.D2 PBMCs depleted of neutrophils (neutrophil depleted). The comparison of IFN-*γ* expression of DX5^+^CD3^−^Ly49G2^+^ NK cells and DX5^+^CD3^−^Ly49C/I^+^ NK cells in control (*n* = 6), PB untreated (*n* = 9), neutrophil rich (*n* = 4), and neutrophil depleted (*n* = 4) was depicted (c). Data are expressed as the means ± SD. ^∗^ *P* < 0.05; ^∗∗^ *P* < 0.01.

We further evaluated whether neutrophils definitely induce a licensing effect of NK cells by using negative selection method with magnet beads. B10.D2 peripheral blood was enriched of CD11b^+^Gr-1^+^ neutrophils that yield to 98.6% purity. Peripheral blood was also depleted of CD11b^+^Gr-1^+^ neutrophils (1.8%) (Figure 4(a)). The expressions of IFN-*γ* in Ly49G2^+^NK cells but not Ly49C/I^+^NK cells of B10 mice at day 2 after intraperitoneal injection of B10.D2 neutrophil-rich group (*n* = 4) were significantly increased than those of PB-untreated group (*n* = 9) (Figures 4(b) and 4(c)). Although we cannot rule out the possibility that other contaminating cells (1.4%) after neutrophil enrichment may still have some effect on the licensing effect, the result of dose-response relationship (Figure 4(c)) may suggest that neutrophils have a licensing effect of NK cells. The effect of elevated IFN-*γ* expression in Ly49G2^+^NK cells induced by PB-untreated group was significantly diminished in neutrophil-depleted group (*n* = 5). These results suggest that NK cells are licensed by presentation of its corresponding NK receptor ligand of neutrophils.

## 4. Discussion

We have shown here that injection of neutrophils induced NK cell licensing via NK receptor ligand interaction, suggesting that licensed (activated) NK cells may potentially induce GVL/GVT effect after allogeneic HSCT. Recently, Jaeger et al. reported direct NK cells/neutrophils interaction [8]. The two cell subsets were localized in close proximity in the red pulp of the spleen or in the medulla next to the lymphatic vessels of the lymph nodes although they did not test the mechanistic significance, including the NK cell licensing. Interestingly, they also described that the* in vitro* coculture of neutrophils with NK cells was not sufficient to restore NK cell function. We confirmed that the* in vitro* coculture of B10 PBMCs with B10.D2 PBMCs resulted in insufficient upregulation of CD107a of NK cells (data not shown). The environment for cross talk and/or cytokine stimulation may be required for the licensing of NK cells. We have successfully promoted a licensing effect by intraperitoneal injection, but not intravenously. The route of injection for licensing may be important because of different access to lymphoid organs. Entry of injected cells into lymph nodes from blood (intravenous injection) occurs through several steps of events that include their rolling on high endothelial venules, arrest, and eventual transmigration into tissues [14]. On the other hand, intraperitoneal injection is an easy way for neutrophils to access several lymphoid tissues [15], resulting in elicitation of a valid interaction between NK cells and neutrophils. Thus, adequate circumstances (lymphoid organs) may be needed to effectively license NK cells.

In the current system, a licensing effect of NK cells was transient (Figure 2). The effect reached its peak at 2 days after injection and restored at 7 days. The reason for the transient effect may be due to a single injection of neutrophils to induce NK cell licensing. Continuous licensing effect may require a constant presentation of NK receptor ligand as previously reported [9, 16]. Constant presentations of NK receptor ligand can be achieved in the setting of allogeneic HSCT for constant supply of cells bearing MHC class I from hematopoietic stem cells. Or long-lasting licensing effects may be attained by multiple neutrophil injections.

Neutrophils are the most abundant blood leukocyte and are key component of the early innate response to pathogens or allogeneic antigens. Previous studies have described the interaction between neutrophils and NK cells and its role in the regulation of their separate activities [8, 17], but few have addressed the receptors involved in this cross talk. Costantini et al. have recently addressed the receptors involved in cross talk between neutrophils and NK cells. They described that human neutrophils can directly cooperate with NK cells present in the blood and tissues to ultimately amplify the production of IFN-*γ* by NK cells upon activation with LPS plus the IL-15/IL-18 combination [18]. On the other hand, they also reported that IL-15 plus IL-18-activated NK cells upmodulate the expression of activation markers such as CD11b, CD64, and CD69 in neutrophils [17]. Thus, interaction between neutrophils and NK cells plays an important role in regulating immune responses.

NK cell licensing by neutrophils* in vivo* may be clinically implicated in the previous studies although precise mechanisms were not elucidated. Antidisialoganglioside GD2 mAb mediates highly efficient antibody-dependent cell-mediated cytotoxicity of high-risk neuroblastoma in the presence of human NK cells and neutrophils* in vitro* [19]. When combined with granulocyte-macrophage colony-stimulating factor (GM-CSF) and interleukin-2 after autologous HSCT, a statistically significant improvement in progression-free survival was observed in neuroblastoma [20]. Interestingly, GM-CSF-induced neutrophil activation* in vivo* is associated with improved patient outcome in neuroblastoma [21]. In addition, Willems et al. reported that recipient leukocyte infusion including neutrophils induces GVL response without GVHD in mice [22]. Allogeneic NK cell alloreactivity seems to have a therapeutic advantage in prevention of relapse of malignancy [2].

In this murine study, we have shown that G-CSF-treated neutrophils as well as purified neutrophils activated NK cells. Murine studies on GVL/GVT effect using licensed NK cells are under way. If successful, it is tempting to speculate that transfer of neutrophils after allogeneic hematopoietic stem cell transplantation may provide graft-versus-leukemia/tumor effects by neutrophil-induced activation of allogeneic NK cells.

## 5. Conclusions

Our data indicate that NK cell function adapted to host environment is licensed by neutrophils through* in vivo* exposure of specific NK receptor ligand. Injection of increasing doses of neutrophils between congenic mice led to increasing expression of CD107a and IFN-*γ*, markers of NK cell activation/licensing. From this finding, it is suggested that one of the mechanisms of interaction between neutrophils and NK cells is licensing. Therefore, a possible benefit of transferring neutrophils after allogeneic stem cell transplantation to augment NK cell mediated graft-versus-tumor effects in humans, if ongoing murine studies on GVL/GVT effects are successful.

## Abbreviations

NK: Natural killer
HSCT: Hematopoietic stem cell transplantation
MHC: Major histocompatibility complex
PBMC: Peripheral blood mononuclear cell
G-CSF: Granulocyte colony-stimulating factor
GM-CSF: Granulocyte-macrophage colony-stimulating factor
IFN-*γ*: Interferon-*γ*
GVL: Graft-versus-leukemia
GVT: Graft-versus-tumor.
